# S100A8 transported by SEC23A inhibits metastatic colonization via autocrine activation of autophagy

**DOI:** 10.1038/s41419-020-02835-w

**Published:** 2020-08-06

**Authors:** Zhiwei Sun, Bin Zeng, Doudou Liu, Qiting Zhao, Jianyu Wang, H. Rosie Xing

**Affiliations:** 1 Institute of Life Sciences, Chongqing Medical University, Chongqing, China; 2 Laboratory of Translational Cancer Stem Cell Research, Chongqing Medical University, Chongqing, China; 3 State Key Laboratory of Ultrasound Engineering in Medicine Co-Founded by Chongqing and the Ministry of Science and Technology, College of Biomedical Engineering, Chongqing Medical University, Chongqing, China

**Keywords:** Metastasis, Macroautophagy

## Abstract

Metastasis is the main cause of failure of cancer treatment. Metastatic colonization is regarded the most rate-limiting step of metastasis and is subjected to regulation by a plethora of biological factors and processes. On one hand, regulation of metastatic colonization by autophagy appears to be stage- and context-dependent, whereas mechanistic characterization remains elusive. On the other hand, interactions between the tumor cells and their microenvironment in metastasis have long been appreciated, whether the secretome of tumor cells can effectively reshape the tumor microenvironment has not been elucidated mechanistically. In the present study, we have identified “SEC23A-S1008-BECLIN1-autophagy axis” in the autophagic regulation of metastatic colonization step, a mechanism that tumor cells can exploit autophagy to exert self-restrain for clonogenic proliferation before the favorable tumor microenvironment is established. Specifically, we employed a paired lung-derived oligometastatic cell line (OL) and the homologous polymetastatic cell line (POL) from human melanoma cell line M14 that differ in colonization efficiency. We show that S100A8 transported by SEC23A inhibits metastatic colonization via autocrine activation of autophagy. Furthermore, we verified the clinical relevance of our experimental findings by bioinformatics analysis of the expression of Sec23a and S100A8 and the clinical-pathological associations. We demonstrate that higher Sec23a and Atg5 expression levels appear to be protective factors and favorable diagnostic (TNM staging) and prognostic (overall survival) markers for skin cutaneous melanoma (SKCM) and colon adenocarcinoma (COAD) patients. And we confirm the bioinformatics analysis results with SKCM biopsy samples.

## Introduction

Metastasis is the main cause of failure of cancer treatment^1–3^. It is characterized by discrete multi-steps: acquisition of the invasive phenotype, local invasion into surrounding stroma and hematogenous circulation, survival in the circulation, extravasation and invasion into distant organs, survival at the secondary site, and colonization to form micro- and macro-metastases^2,4–6^. The last stage, i.e., the colonization of invaded tumor cells, is regarded most rate-limiting for metastasis. Colonization is also a multi-step process^2,5,6^: first, the extravasated tumor cells enter a period of dormancy to withstand the restrains posted by the foreign microenvironment and immune surveillance. Second, survived tumor cells activate clonogenic proliferation in order to form micro-metastases. Finally, upon establishing favorable tumor microenvironment, micro-metastases will develop into clinically detectable macro-metastases.

Interactions between the tumor cells and their microenvironment play a vital role in the entire metastatic cascade, especially in the colonization at the distant site^6–8^. However, mechanistic understanding of the interactions between the tumor cells and their microenvironment at the site of metastasis has been largely focused on how tumor cells will overcome the restrains of the foreign microenvironment to develop into micro- and macro-metastatic lesions, little is known about whether tumor cells exert self-restrains upon extravasation.

The secretome of tumor cells can effectively reshape the tumor microenvironment via autocrine regulation of tumor cells or paracrine interactions with the stromal cells. SEC23A is an important constituent of coat protein complex II (COPII) that is responsible for the transportation of secreted proteins from rough endoplasmic reticulum to Golgi apparatus^9–11^. And SEC23A has been reported to participate in chondrogenesis^12,13^ and suppress tumor metastasis^14–17^ by regulating tumor cell protein secretion, i.e., the secretome. Our previous study has characterized alterations in the composition of the SEC23A secretome upon Sec23a silencing in M14 human melanoma cells and identified S100A8 on the list of the significantly decreased secreted proteins^18^. S100A8 is a multi-functional protein^19–21^. It is a calcium-binding protein and polymerizes with S100A9 to form calprotectin for metals iron, manganese and zinc detention via chelation. Secreted S100A8 may regulate inflammatory response within the tumor microenvironment. However, the role of S100A8 in metastasis has not been characterized prior to this study.

Autophagy is an evolutionarily conserved biological process of energy metabolism^22,23^. By degrading intracellular organelles and proteins, autophagy provides cells with biochemical reaction substrates for the maintenance of homeostasis under nutrient deprivation or other stressful conditions^24–27^. Both the anti- and pro-metastatic roles of autophagy have been reported and appear to be context and stage-dependent^28–31^: in the initial stage of metastasis, autophagy may inhibit metastasis by promoting the release of anti-metastatic immunomodulatory factors^29,32–34^. Once tumor cells enter hematogenous circulation, autophagy may promote metastasis by protecting the circulating tumor cells from anoikis^29,35–37^. During colonization at the metastatic site, the role of autophagy becomes intricate. On the one hand, autophagy keeps the extravasated tumor cells in the dormancy stage thus prevents proliferation and colonization^29,38,39^. On the other hand, once micro-metastases are established, autophagy switches to promote macro-metastases via helping tumor cells adapt to the foreign microenvironment^29,40,41^.

In the present study, we focus on uncovering mechanisms underlying autophagic regulation of the colonization step of metastasis. We employed a paired of lung-derived oligometastatic cell line (OL) and the homologous polymetastatic cell line (POL) from human melanoma cell line M14 that we previously developed and characterized^18,42^. We have elucidated a new mechanism by which the secretome of tumor cells hinders metastasis through activation of autophagy. Specifically, S100A8 transported by SEC23A inhibits metastatic colonization via autocrine activation of autophagy in extravasated tumor cells. Furthermore, Sec23a and Atg5 are favorable diagnostic and prognostic markers for human melanoma and colon cancer.

## Materials and methods

### Cell lines and cell culture

M14 cells were kindly provided by Dr. Robert Hoffman (University of California San Diego). By isolation of M14 cells from the lung of mice exhibited oligometastasis or polymetastasis, respectively, in vivo, followed by three rounds of in vivo validation, OL and POL cell lines from human melanoma cancer cell line M14 were established^18,42^. M14, OL and POL cell lines and its derivative cell lines were cultured in DMEM (Hyclone) supplemented with 10% fetal bovine serum (FBS) (Gibco) and 1% penicillin-streptomycin (Hyclone).

### Lentivirus production

The sequence for the sh-RNAs targeting Sec23a was 5′-GGAAGCTACAAGAATGGTTGT-3′. Sec23a overexpression (OE) plasmid pLVX-Puro-mRuby-Sec23a (Plasmid #36158) was provided by Addgene. The lentivirus particles of shSec23a and Sec23a-OE were prepared by Sangon Biotech Co. The lentivirus particles of shAtg5 LV-ATG5-RNAi(9513-1) were purchased from GENECHEM. The sequence for the sh-RNAs targeting S100A8 was 5′-CCUGAAGAAAUUGCUAGAGTT-3′ and the lentivirus particles of shS100A8 were prepared by GENECHEM.

### Autophagy inhibitors and activator

3-MA (M9281) was purchased from Sigma. Baf-A1 (S1413) and Rapamycin (S1039) were purchased from Selleck.

### Reverse transcription and quantitative real-time polymerase chain reaction (RT-qPCR)

Total RNAs were isolated using Trizol (Takara, Japan) and reverse-transcribed into cDNA using PrimeScript RT Master Mix (Takara, Japan). RT-qPCR was performed using SYBR Green Real-time PCR Master Mix kit (Takara, Japan) according to the manufacturer’s instructions. The following PCR condition was used on the Light Cycler: 39 cycles of 95 °C for 30 s, 95 °C for 5 s, followed by 60 °C for 30 s in a 10 µl reaction volume. Relative expression was normalized to that of GAPDH internal control. The forward primer sequences and reverse primer sequences of Sec23a, Atg5, S100A8, and GAPDH were AGTGGCGGAAGTCAGGATAC and GGCATTGGAAATCTGGAGTG; CTCTGCAGTGGCTGAGTGAA and TCAATCTGTTGGCTGTGGGA; CCGAGCTGGAGAAAGCCTTG and AGGTCATCCCTGTAGACGGC; AGAAGGCTGGGGCTCATTTG and AGGG GCCATCCACAGTCTTC.

### Western blot (WB) analysis

Cells were lysed in SDS lysis buffer (Beyotime, P0013G) containing 1% protease inhibitor PMSF (Beyotime, ST506). Extracted protein concentration was determined using the BCA protein assay kit (Beyotime, P0012S) and stored at −80 °C. 20 μg of each protein sample were separated by electrophoresis with 12% polyacrylamide gels and transferred to polyvinylidene fluoride (PVDF) membranes (Millipore, IPVH00010). After blocking, the membranes were incubated with appropriate primary antibodies and secondary antibodies. The primary antibody of SEC23A (#8162, 1:800) was purchased from Cell Signaling Technology^®^. The primary antibody of LC3B (ab192890, 1:1000) and P62 (ab207305, 1:2000) were purchased from Abcam. The primary antibody of ATG5 (10181-2-AP, 1:1000), BCL2 (12789-1-AP, 1:500), BECLIN1 (11306-1-AP, 1:500) and TUBULIN (10068-1-AP, 1:2000) were purchased from Proteintech. The primary antibody of S100A8 (HPA024372, 1:500) was purchased from Sigma. The secondary antibody of anti-Rabbit (SA00001-2, 1:2000) was purchased from Proteintech.

### Immunoprecipitation analysis

Cells were lysed at 4 °C in ice-cold lysis buffer (Beyotime) and cell pallet prepared by centrifugation. Immunoprecipitation was performed using BECLIN1 antibody, and the BECLIN1 protein complexes were captured with protein A+G agarose beads (Beyotime). Then the beads-bound proteins were eluted by boiling in SDS sample buffer and subjected to polyacrylamide gel electrophoresis and analyzed by WB analysis.

### Autophagy flux analysis of LC3B puncta

Adenovirus expressing mCherry-GFP-LC3B fusion protein (Ad-mCherry-GFP-LC3B, C3011) was purchased from Beyotime. Cells were plated in 6-well plates and allowed to reach 50–70% confluence at the time of Ad-mCherry-GFP-LC3B transfection. Adenoviral infection was performed according to the manufacturer’s instructions. The presence of mRFP-LC3B puncta indicated the autolysosomes in red fluorescent images.

### Transmission electron microscopy (TEM) analysis of autolysosomes

Cells were harvested and centrifuged at 1200 rpm/min for 10 min. Cell pellet was fixed with 4% glutaraldehyde and 1% osmium tetroxide. Thereafter the cell pellet was dehydrated in a graded series of alcohol and acetone and followed by embedment in Epon 816 (Electron Microscopy Sciences). Ultrathin sections were cut by a Leica ultramicrotome (Leica Microsystems) and stained with uranyl acetate and lead citrate. TEM was conducted by JEM-1400Plus transmission electron microscope (JEOL Ltd).

### Transwell migration and invasion assay

Transwell migration and invasion assay were conducted as we previously reported^18^. Briefly, 8 μm pore size transwell inserts and Matrigel for invasion assay were purchased from BD. 300 μl serum-free medium with 5 × 10^4^ tumor cells was seeded into the upper chamber and 800 μl medium with 20% FBS was added into the lower chamber. The migrated or invaded tumor cells on the lower surface of transwell membrane were fixed with cold methanol and stained with crystal violet.

### Soft agar colony formation assay

Colony formation assay was conducted as we previously reported^18^. Briefly, tumor cells in DMEM containing 2% agar and 10% FBS were plated into 6-well plates and coated with DMEM containing 0.5% agar. Cell colonies that consisted of more than 50 cells were stained with crystal violet and counted.

### TCGA database analysis

mRNA expression of Sec23a and Atg5 in human skin cutaneous melanoma and colon adenocarcinoma were analyzed by TCGA Research Network (http://cancergenome.nih.gov). To analyze the survival of patients with skin cutaneous melanoma and colon adenocarcinoma, patient samples were analyzed by OncoLnc (http://www.oncolnc.org).

### Animal experiments

Animal experiments was conducted as we previously reported^18^. All NOD/SCID mice used in the study were obtained from the core facility of Experimental Animal Centre in Chongqing Medical University. Each NOD/SCID mouse was transplanted with 5 × 105 tumor cells via tail intravenous injection and dissected on day 27 post tumor cell inoculation. Whole-lung bright, fluorescent and H&E images were used to examine and quantify lung-derived metastatic nodules.

### Compliance with ethics guidelines

All animal work was conducted in accordance with an approved protocol and carried out in accordance with the institutional animal welfare guidelines of the Chongqing Medical University. All of the formalin fixed paraffin embedded clinical specimens were obtained from department of pathology in the affiliated hospital of southwest medical university through a protocol approved by the institutional ethics committee.

### Statistical analysis

All data were analyzed by Student’s independent *t*-test of variance using GraphPad Prism software and presented as mean ± SEM. Differences were considered statistically significant when *P < 0.05, **P < 0.01 and ***P < 0.001.

## Results

### Sec23a inhibits the metastasis of melanma cells in vitro and in vivo

**Fig. 1:**
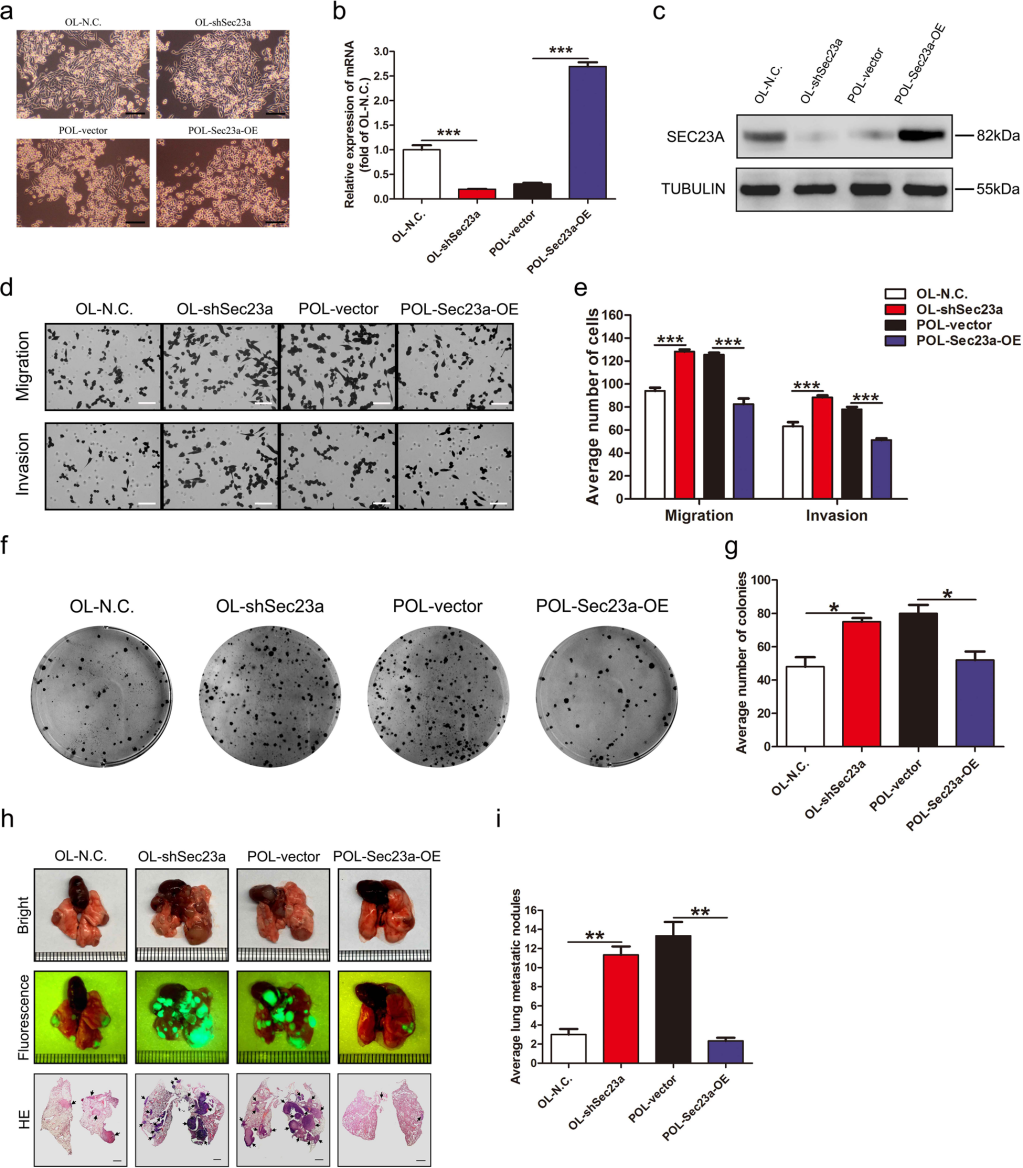
Sec23a inhibits lung-derived metastasis of melanoma cells. **a** Representative images of OL-N.C., OL-shSec23a, POL-vector, and POL-Sec23a-OE cells. Scale bars, 200 μm. **b**, **c** RT-qPCR and western blot were performed to confirm gene manipulation of Sec23a expression in OL and POL. ***P < 0.001. **d**, **e** Representative images and quantification of transwell migration and invasion assay. Scale bars, 60 μm. ***P < 0.001. **f**, **g** Representative images and quantification of colony formation assay. *P < 0.05. **h** Lung-derived metastatic nodules were shown by whole-lung bright, fluorescent and H&E images. Scale bars, 1 mm. **i** Quantification of lung-derived metastatic nodules. **P < 0.01.

In our previous study, a set of paired lung-derived oligometastatic cell model (OL) and the homologous polymetastatic cell model (POL) from human melanoma cancer cell line M14 were established and characterized^42^. Sec23a, a confirmed gene target of miR-200c, was shown to mediate the oligometastatic to polymetastatic progression that differ in metastatic colonization efficiency^18^. To further investigate the mechanism of the inhibitory role of Sec23a in tumor metastasis, stable Sec23a interference in OL or overexpression in POL was achieved by lentivirus infection (Fig. 1a) and confirmed by RT-qPCR (Fig. 1b) and western blot (Fig. 1c), respectively. Consistent with our previous report^18^, stable Sec23a interference markedly enhanced the migration and invasion ability of OL-shSec23a cells in comparison with the OL-N.C. control cells. Inversely, stable Sec23a overexpression significantly impaired the migration and invasion ability of POL-Sec23a-OE cells in comparison with the POL-vector control cells (Fig. 1d–e). The colonization capacity in vitro of tumor cells with altered Sec23a expression was evaluated via soft agar colony formation assay. The results showed that colony formation in vitro was enhanced in OL-shSec23a cells while inhibited in POL-Sec23a-OE cells (Fig. 1f, g). To evaluate the effect of Sec23a expression status on metastatic colonization efficiency in vivo, we inoculated OL-shSec23a, POL-Sec23a-OE, and control tumor cells to NOD/SCID mice by tail vein injection. Mice injected with OL-shSec23a cells suffered with significantly enhanced lung metastatic tumor burden in comparison with that injected with OL-N.C. cells. In contrast, in mice injected with POL-Sec23a-OE cells, lung metastatic tumor burden was significantly attenuated in comparison with that injected with POL-vector cells (Fig. 1h, i). In addition, Sec23a had been shown to have no significant effect on cell proliferation and viability in our previous research^18^. These results show that Sec23a inhibits metastatic colonization of M14 cells both in vitro and in vivo.

### Sec23a activates autophagy in melanoma cells

**Fig. 2:**
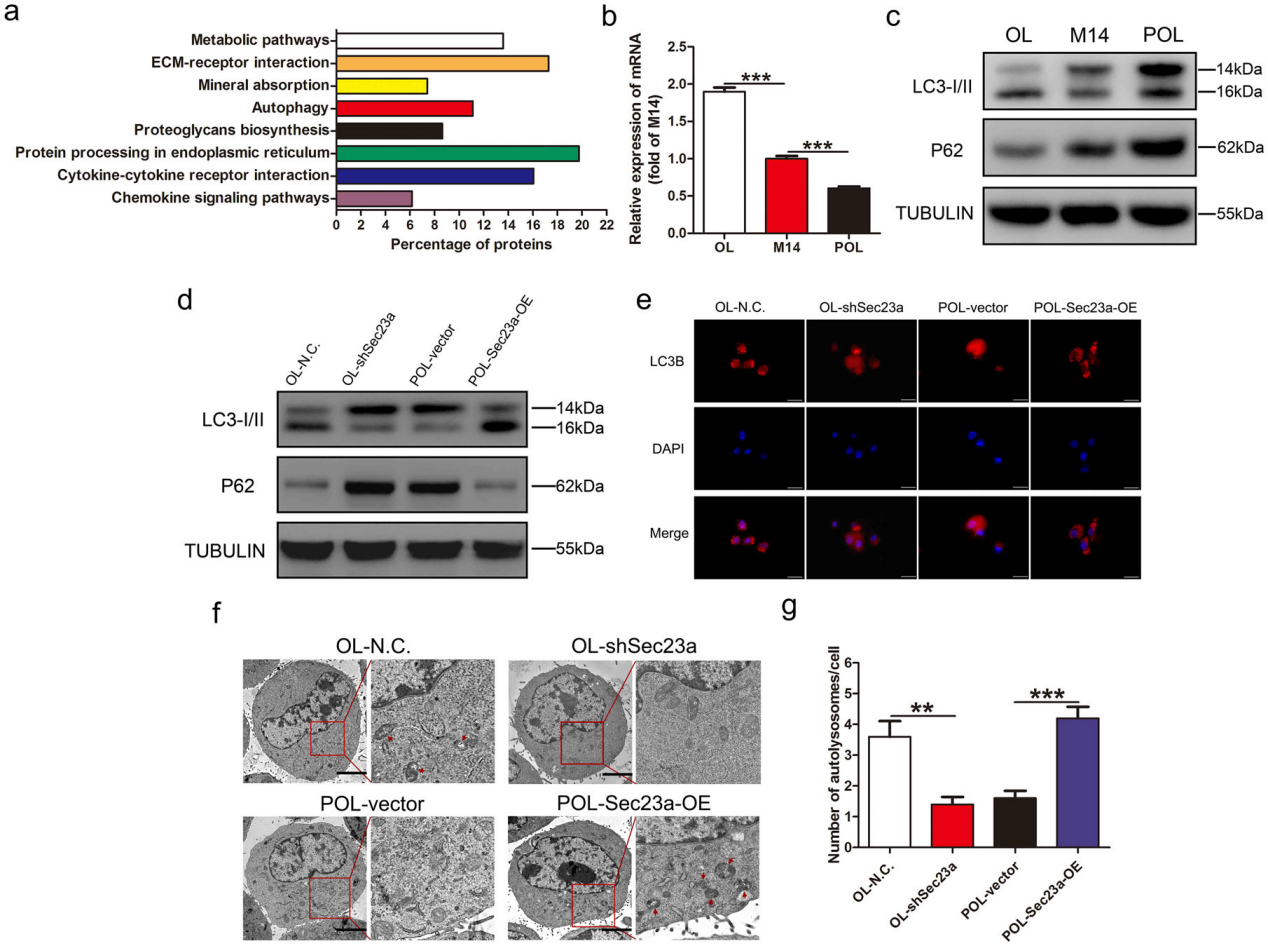
Sec23a activates autophagy in melanoma cells. **a** Functional cluster analysis of the decreased secreted proteins after Sec23a interference. Sec23a expression was stablely knocked down in OL cells via lentiviral infection. Conditioned media from OL-N.C. and OL-shSec23a cells were concentrated and freeze-dried, followed by protein quantization and SDS-PAGE electrophoresis. Thereafter, protein samples were subjected to reductive alkylation, enzymatic hydrolysis, and labeled with Tandem Mass Tags. Samples were loaded onto NanoLC trap column and data acquisition was performed with Q-Exactive System fitted with a Nanospray. The spectral data files were tanalyzed using the SEQUEST algorithm available in Proteome Discoverer 1.4 software. **b** Sec23a mRNA abundance in OL, M14, and POL cells measured by real-time quantitative PCR. **c** Western blot analysis for LC3I/II and P62 in M14 and M14 derivative OL and POL cells. **d** Western blot analysis for LC3I, LC3II, and P62 in OL-N.C., OL-shSec23a, POL-vector, and POL-Sec23a-OE cells. **e** OL-N.C., OL-shSec23a, POL-vector, and POL-Sec23a-OE cells expressed mRFP-LC3B fusion protein via adenovirus infection. Representatives of LC3B-positive puncta images were shown. Scale bars, 20 μm. **f** Transmission electron microscopy of OL-N.C., OL-shSec23a, POL-vector and POL-Sec23a-OE cells. Autolysosomes indicated by arrowheads. Scale bars, 5 μm. **g** Mean number of detectable autolysosomes in each tumor cell, counted on transmission electron microscopy images. **P < 0.01, ***P < 0.001.

In our previous study, we conducted mass spectrometry (MS) analysis to identify the significantly decreased secreted proteins upon Sec23a interference^18^. To elucidate the mechanisms underlying the inhibitory role of Sec23a in metastasis, a functional cluster analysis of the decreased secreted proteins was carried out. We noticed with great interest that some of the decreased secreted proteins were involved in autophagy, indicating that Sec23a may regulate autophagy (Fig. 2a), a function of Sec23a that has not been identified. The expression level of Sec23a in OL cells was higher than that in M14 cells, and the expression level of Sec23a in POL cells was lower than that in M14 cells (Fig. 2b). In addition, compared with parental M14 cells, OL cells with higher Sec23a expression possessed enhanced basal level of autophagy and whereas in POL cells with lower Sec23a expression than M14 cells, basal autophagy level was suppressed (Fig. 2c).

To investigate whether Sec23a may regulate autophagy, three classic assays commonly used in autophagy research were used to characterize autophagy status when Sec23a expression was altered by gene manipulation: (1) the protein level of LC3 and P62 by western blot (WB), (2) mRFP-LC3B punta by immunofluorescence (IF), and (3) cytoplasmic accumulation of autolysosomes by transmission electron microscopy (TEM) analysis (see “Materials and methods” section). WB analysis revealed a positive relationship between Sec23a expression and autophagy., i.e., autophagy was impaired upon Sec23a interference and enhanced with Sec23a overexpression (Fig. 2d). Sec23a activation of autophagy was confirmed by the increased mRFP-LC3B punta (Fig. 2e). The formation of autolysosomes and autolysosomes analyzed by TEM were markedly reduced in tumor cells with Sec23a interference and augmented in tumor cells with Sec23a overexpression (Fig. 2f, g). These results provide sufficient evidence that Sec23a augments autophagy in M14 cells.

### Sec23a inhibits the metastasis of melanma cells through activation of autophagy

**Fig. 3:**
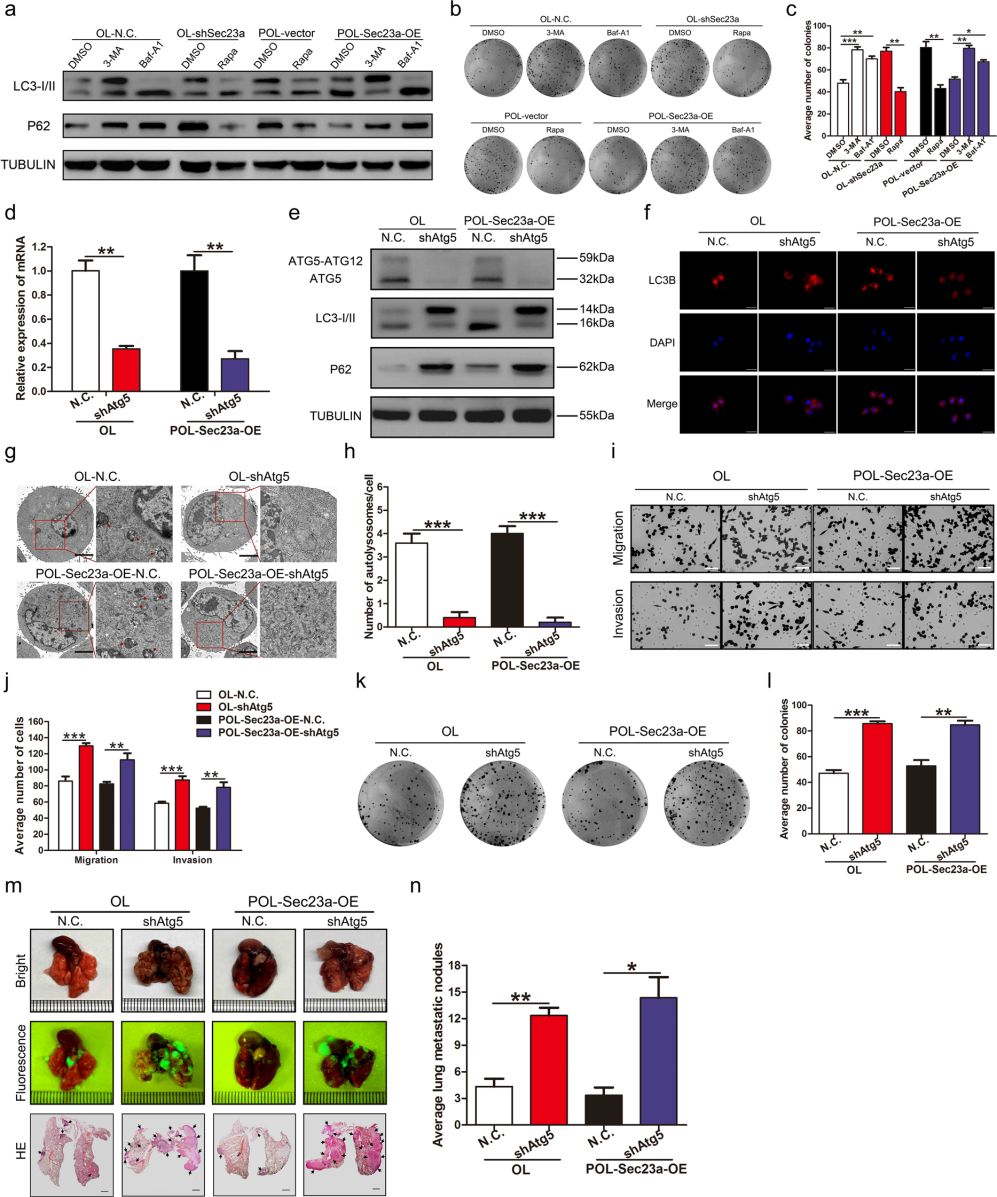
Autophagy inhibits metastatic colonization of melanoma cells. **a** Western blot analysis for LC3I, LC3II and p62 in tumor cells treated with 3-MA, Baf-A1, or Rapa. **b**, **c** Representative images and quantification of colony formation assay of tumor cells treated with 3-MA, Baf-A1, or Rapa. *P < 0.05, **P < 0.01, ***P < 0.001. **d** RT-qPCR was performed to confirm Atg5 interference in OL and POL-Sec23a-OE cells. **P < 0.01. **e** Western blot analysis for ATG5, LC3I/II and p62 in OL and POL-Sec23a-OE cells with Atg5 interference. **f** Representative images of LC3B-positive puncta in tumor cells with Atg5 interference. Scale bars, 20 μm. **g** Transmission electron microscopy of tumor cells with Atg5 interference. Autolysosomes indicated by arrowheads. Scale bars, 5 μm. (**h**) Quantification of detectable autolysosomes after Atg5 interference, counted on transmission electron microscopy images. ***P < 0.001. **i**, **j** Representative images and quantification of transwell migration and invasion assay. Scale bars, 60 μm. **P < 0.01, ***P < 0.001. **k**, **l** Representative images and quantification of colony formation assay. **P < 0.01, ***P < 0.001. **m** Lung-derived metastatic nodules were shown by whole-lung bright, fluorescent and H&E images. Scale bars, 1 mm. **n** Quantification of lung-derived metastatic nodules. *P < 0.05, **P < 0.01.

Given the data showing that Sec23a inhibited metastatic colonization and activated autophagy in melanoma cells, we next evaluated whether anti-metastatic colonization effect of Sec23a is dependent on autophagy using M14 derivative OL and POL cells. Firstly, autophagy activity was either inhibited by synthetic inhibitors (3-MA and Baf-A1) or stimulated with Rapa and assessed by three assays as described above (Fig. 3a; Supplementary Fig. S1). The mechanism underlying Baf-A1 inhibition of autophagy is blocking the binding of autophagosomes to lysosomes thus to inhibit the formation of autolysosomes. When autophagy activity was inhibited by Baf-A1, punctate LC3B was still detectable in the autophagy flux assay (Supplementary Fig. S1). And Baf-A1 treatment decreased the total amount of autolysosomes under electron microscopy observation (Supplementary Fig. S1). Colony formation of OL- and POL-M14 cells treated with autophagy inhibitors 3-MA and Baf-A1 was increased, whereas it was decreased with Rapa treatment (Fig. 3b, c). The Transwell migration and invasion assay showed that 3-MA and Baf-A1 augmented the migration and invasion capacity of melanoma cells while Rapa attenuated both (Supplementary Fig. S2).

Then autophagy was inhibited by stable Atg5 interference in cells that expressed high levels of Sec23a (Fig. 3d, e). Autophagic flux assay and TEM analysis were conducted to confirm the attenuated autophagy activity upon Atg5 silencing (Fig. 3f–h). In vitro, Atg5 interference reversed the inhibitory effect of high Sec23a expression on migration, invasion and colonization capacity (Fig. 3i–l). In vivo, NOD/SCID mice injected with tumor cells with stable Atg5 interference developed significantly more extensive macroscopic lung metastases (Fig. 3m, n). These data showed convincingly that the inhibitory effect of Sec23a on M14 lung metastatic colonization requires activation of autophagy.

### S100A8 transported by SEC23A augments autophagy in melanoma cells

**Fig. 4:**
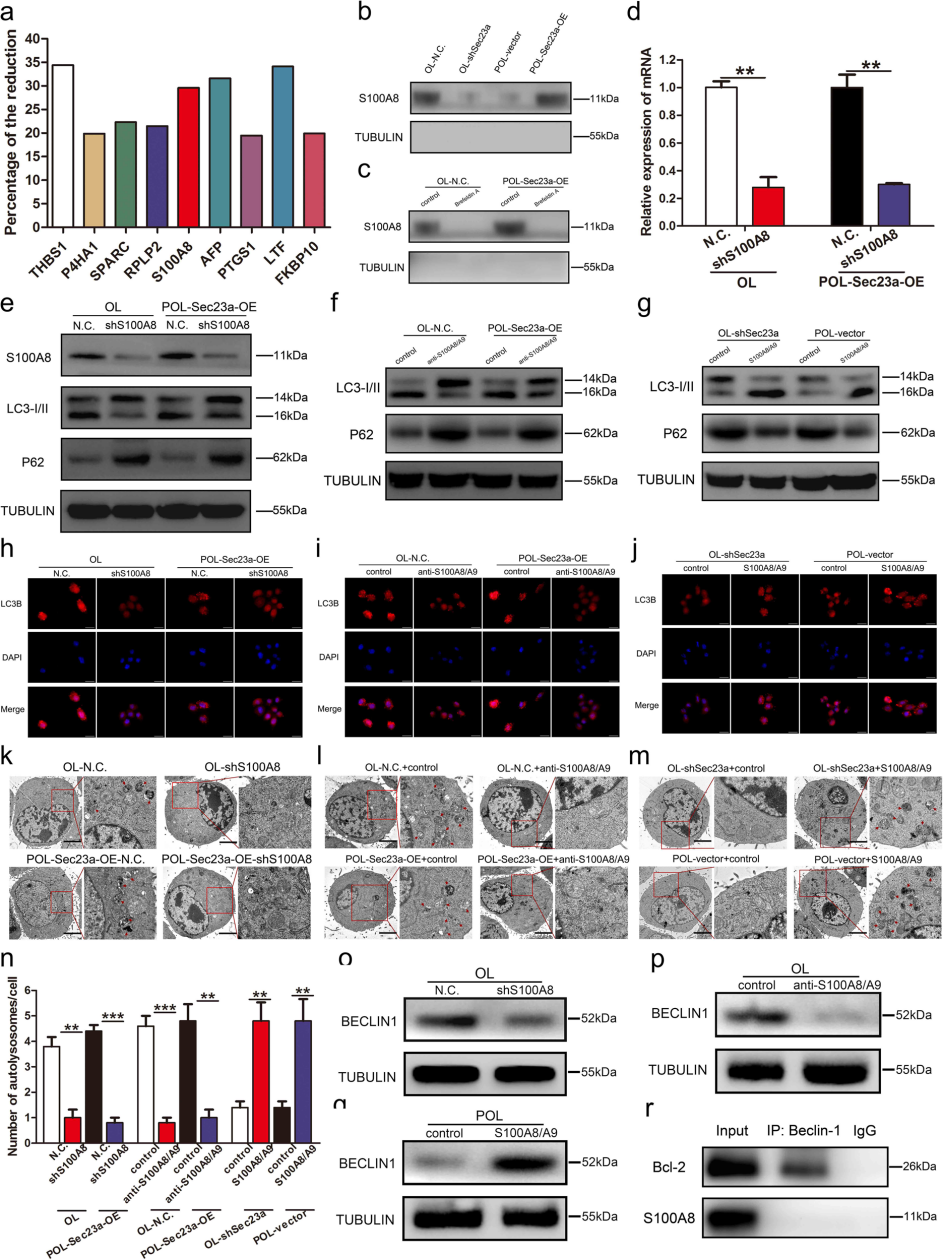
S100A8 transported by SEC23A augments autophagy in melanoma cells. **a** Quantitative analysis of autophagy-associated secreted protein transported by SEC23A. **b** Western blot analysis for S100A8 in the conditional medium of OL-N.C., OL-shSec23a, POL-vector, and POL-Sec23a-OE cells. **c** Western blot analysis for S100A8 in the conditional medium of tumor cells treated with Brefeldin A. **d** RT-qPCR was performed to confirm S100A8 interference in OL and POL-Sec23a-OE cells. **P < 0.01. **e** Western blot analysis for S100A8, LC3I/II and p62 in OL, and POL-Sec23a-OE cells with S100A8 interference. **f**, **g** Western blot analysis for LC3I, LC3II, and P62 in tumor cells treated with anti-S100A8/A9 antibody or recombinant S100A8/A9 protein dimer. **h**–**j** LC3B-positive puncta detected in tumor cells after S100A8 interference, anti-S100A8/A9 antibody treatment, or recombinant S100A8/A9 protein dimer treatment. Scale bars, 20 μm. **k**–**n** Detection and quantification of autolysosomes in tumor cells after S100A8 interference, anti-S100A8/A9 antibody treatment or recombinant S100A8/A9 protein dimer treatment. Autolysosomes indicated by arrowheads in TEM images. Scale bars, 5 μm. **P < 0.01, ***P < 0.001. **o**–**q** Western blot analysis for BECLIN1 in tumor cells after S100A8 interference, anti-S100A8/A9 antibody treatment or recombinant S100A8/A9 protein dimer treatment. **r** Co-IP assay to detect interaction between S100A8 and BECLIN1.

Based on our previous MS and functional cluster analysis, some of the decreased secreted proteins after Sec23a interference were involved in autophagy^18^. We performed a confirmatory quantitative analysis of secreted proteins that may be related to autophagy and found a promising candidate S100A8 (Fig. 4a), a known stimulator of autophagy^43–45^.

To confirm that S100A8 secretion is SEC23A-dependnet in our M14 derivative cell lines, conditional medium of OL-N.C., OL-shSec23a, POL-vector, and POL-Sec23a-OE cells were collected and concentrated for WB analysis (see “Materials and methods” section). WB showed that the quantity of secreted S100A8 was Sec23a-dependent, i.e., it was decreased upon Sec23a interference and increased upon Sec23a overexpression (Fig. 4b). Thereafter, OL-N.C. and POL-Sec23a-OE cells were treated with Brefeldin A, a specific inhibitor of protein transport between the endoplasmic reticulum and the Golgi apparatus. Brefeldin A treatment dramatically decreased the quantity of secreted S100A8 (Fig. 4c). These results demonstrate that secreted S100A8 is transported by SEC23A.

Then stable S100A8 interference was achieved by lentivirus infection in cells that expressed high levels of Sec23a (Fig. 4d, e). WB analysis of P62 and LC3 lipidation showed that S100A8 interference significantly inhibited autophagy activity (Fig. 4e). The effect of secreted S100A8 on autophagic activity was verified by using neutralizing anti-S100A8/A9 antibody and recombinant S100A8/A9 protein dimer (Fig. 4f–n).

S100A8 has been reported to promote autophagy either through the cross-talk between mitochondria and lysosomes via ROS, or through the formation of BECLIN1-PI3KC3 complex^43–45^. We first examined whether BECLIN1 expression could be altered by S100A8 interference, anti-S100A8/A9 antibody, and recombinant S100A8/A9 protein dimer. We observed coordinated changes in BECLIN1 expression and S100A8 expression in M14 derivative cell lines (Fig. 4o–q), consistent with literature findings^43–45^. This observation suggests that S100A8 may also augment the formation of autophagy initiation complex BECLIN1–PI3KC by increasing BECLIN1 expression. We next examined whether there is a direct interaction between S100A8 and BECLIN1 by co-immunoprecipitation (Co-IP) assay. No direct interaction between S100A8 and BECLIN1 was found (Fig. 4r). Thus, regulation of BECLIN1 by S100A8 is indirect and merits future investigation. These results collectively demonstrate that S100A8 transported by SEC23A augments autophagy via promoting autophagy initiation activator BECLIN1 expression in melanoma cells.

### S100A8 acts downstream of Sec23a to attenuate lung metastatic colonization of melanoma cells

**Fig. 5:**
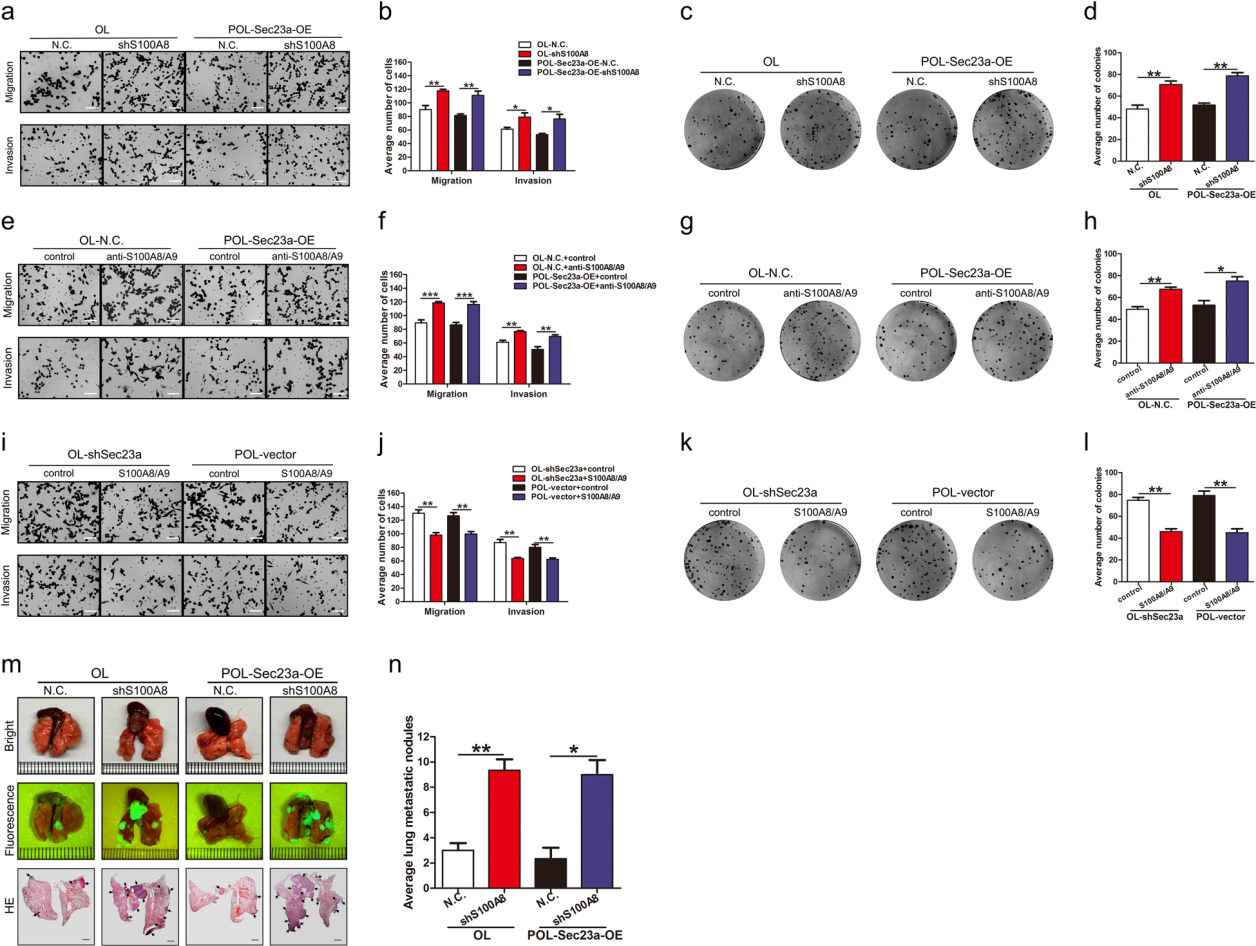
S100A8 acts downstream of Sec23a to attenuate lung metastatic colonization of melanoma cells. **a**, **b** Representative images and quantification of transwell migration and invasion assay of tumor cells with S100A8 interference. Scale bars, 60 μm. *P < 0.05, **P < 0.01. **c**, **d** Representative images and quantification of colony formation assay of tumor cells with S100A8 interference. **P < 0.01. **e**, **f** Representative images and quantification of transwell migration and invasion assay of tumor cells treated with anti-S100A8/A9 antibody. Scale bars, 60 μm. **P < 0.01, ***P < 0.001. **g**, **h** Representative images and quantification of colony formation assay of tumor cells treated with anti-S100A8/A9 antibody. *P < 0.05, **P < 0.01. **i**, **j** Representative images and quantification of transwell migration and invasion assay of tumor cells treated with recombinant S100A8/A9 protein dimer. Scale bars, 60 μm. **P < 0.01. **k**, **l** Representative images and quantification of colony formation assay of tumor cells treated with recombinant S100A8/A9 protein dimer. **P < 0.01. **m** Lung-derived metastatic nodules were shown by whole-lung bright, fluorescent and H&E images. Scale bars, 1 mm. **n** Quantification of lung-derived metastatic nodules. *P < 0.05, **P < 0.01.

Next, we studied the effect of S100A8 on the metastatic characteristics of melanoma cells. S100A8 interference and anti-S100A8/A9 antibody treatment significantly improved the migration, invasion and metastatic colonization capacity of OL and POL-Sec23a-OE cells both in vitro (Fig. 5a–h). In contrast, recombinant S100A8/A9 protein dimer treatment produced the opposite effects in in vitro (Fig. 5i–l). In vivo, NOD/SCID mice injected with tumor cells with stable S100A8 interference developed significantly more extensive macroscopic lung metastases (Fig. 5m, n). This set of results confirm that S100A8 acts downstream of Sec23a to inhibit lung metastatic colonization.

### Low expression of Sec23a and Atg5 is associated with advanced TNM stages and poor prognosis in human skin cutaneous melanoma and colon adenocarcinoma

**Fig. 6:**
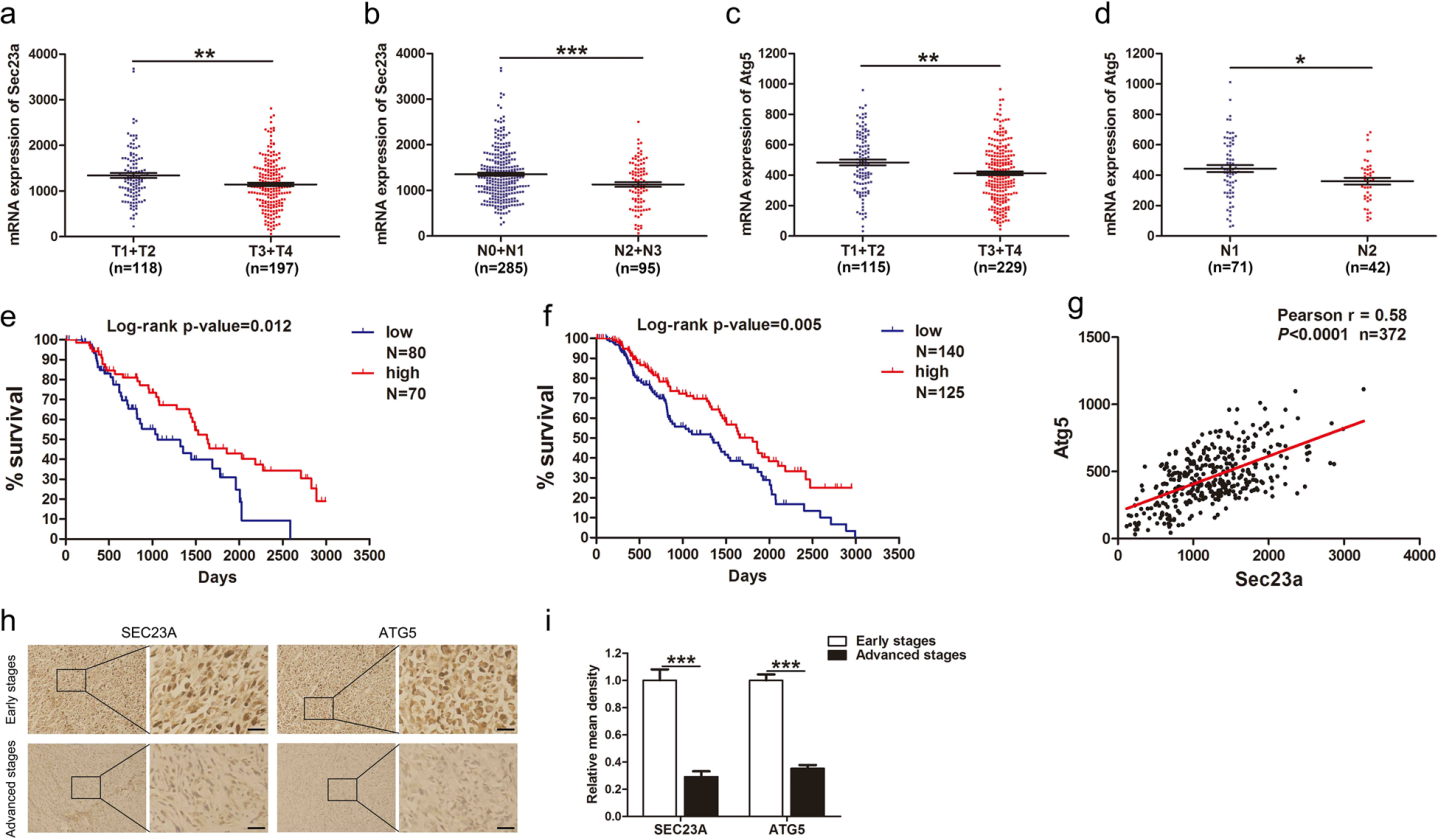
Low expression of Sec23a and Atg5 is associated with TNM stages and poor prognosis in human skin cutaneous melanoma. **a**, **b** Correlation between Sec23a expression and tumor stages in SKCM patients using the TCGA database. **P < 0.01, ***P < 0.001. **c**, **d** Correlation between Atg5 expression and tumor stages in SKCM patients using the TCGA database. *P < 0.05, **P < 0.01. **e**, **f** Low Sec23a and Atg5 expression levels indicated poor overall survivals for SKCM patients using Kaplan–Meier plots analysis. **g** Correlation between Sec23a and Atg5 expression levels in SKCM patients using Spearman test. **h**, **i** Biopsy samples of early and advanced SKCM patients were subjected to IHC analysis to detect different SEC23A and ATG5 expression levels. Scale bars, 30 μm. ***P < 0.001.

We performed clinical data analysis using the TCGA (The Cancer Genome Atlas) database to explore the clinical relevance of our experimental results. Sec23a and Atg5 expression level were both significantly lower in skin cutaneous melanoma (SKCM) patients with advanced primary tumors and regional lymph node metastasis in comparison with SKCM patients of early stages (Fig. 6a–d). Kaplan–Meier plots analysis revealed that SKCM patients with high Sec23a or Atg5 expression levels had significantly better overall survivals in comparison with patients with low Sec23a or Atg5 expression level (Fig. 6e, f). Furthermore, Sec23a expression was positively correlated with Atg5 expression in SKCM patients (Fig. 6g). To confirm bioinformatics analysis results, we performed immunohistochemical (IHC) analysis of SEC23A and ATG5 expression using early and advanced SKCM biopsy samples. SEC23A and ATG5 expression levels of the advanced SKCM biopsy samples were distinctly lower than that of the early SKCM biopsy samples (Fig. 6h, i, *n* = 26). Then we expanded TCGA analysis to other cancers. In colon adenocarcinoma (COAD), similar with SKCM, there was also an inverse-relationship between Sec23a and Atg5 expression and the TNM stages (Supplementary Fig. S3a–f) and overall survivals (Supplementary Fig. S3g, h). Sec23a and Atg5 expression levels were positively correlated in COAD patients (Supplementary Fig. S3i). These observations collectively demonstrate the clinical relevance of the new mechanism of “Sec23a-S100A8-autophagy axis” that we have identified in this study with respect to cancer progression and prognosis.

## Discussion

Although the interactions between the tumor cells and their microenvironment in the entire metastatic cascade have long been appreciated^6–8^, little is known about whether tumor cells can exert self-restrains upon extravasation at the distant sites. As the consensus of the context-dependency of autophagy in metastasis has been reached, studies on the involvement of autophagy metastatic colonization have yielded contradictory findings^46–50^.

Korpal et al.^14^ first reported Sec23a regulated secretome in mediating the pro-metastatic activity of miR-200c in breast cancer cell line. Our prior study has characterized the anti-metastatic effect of Sec23a at the colonization step in M14 melanoma derivative cell lines that differ in colonization efficiency^18^. In the present study, we focus on uncover mechanisms underlying autophagy regulation of the colonization step of metastasis and have made the following novel findings that identified “SEC23A-S1008-BECLIN1-autophagy axis” in the autocrine inhibition of lung metastatic colonization.

The contradictory findings regarding the role of autophagy in metastatic colonization partially resulted from the different experimental cell models. As some studies used highly metastatic tumor cell lines, others used derivative cell lines with enhanced metastatic capability that were derived from low-metastatic cancer cell lines. More importantly, stable and well-characterized cell models that differ in colonization capability were lacking, prohibiting mechanistic inquisition of the role of autophagy in metastatic colonization and proper interpretation of the experimental findings.

To overcome these limitations, we employed a set of paired stable lung-derived oligometastatic cell line OL and the homologous polymetastatic cell line POL from human melanoma cell line M14 that we previously developed and characterized^42^. Although both OL and POL were metastatic, POL cells were more efficient in colonization^18,42^. We have elucidated a new mechanism by which the secretome of tumor cells hinders metastasis through autocrine activation of autophagy that is exemplified by the “SEC23A-S100A8-BECLIN1-autophagy axis”. The intricate role of autophagy in the colonization of invaded tumor cells may also contribute to the reported discrepancies. On the one hand, autophagy keeps the newly extravasated tumor cells in the dormancy stage thus prevents proliferation and colonization^29,38,39^. On the other hand, once micro-metastases are established, autophagy switches to promote macro-metastases via helping tumor cells adapt to the stressful foreign microenvironment^29,40,41^. In the present study, we show that tumor cells may exploit autophagy to exert self-restrain for proliferation before the favorable tumor microenvironment is established.

Prior to this study, S100A8 has been reported to promote autophagy in cancer cells through the cross-talk between mitochondria and lysosomes via ROS, or through the activation of the autophagy initiation complex BECN1-PI3KC3^43–45^. In the latest study, SEC23B, another homolog protein of SEC23 subfamily, has been reported to activate autophagy^51^. Under nutrient deprivation condition, SEC23B and COPII vesicles cease the transportation of secreted proteins. Instead, they serve as precursors for LC3B lipidation in order to provide membrane sources for the autophagosome^51^. Nevertheless, regulation of autophagy by the SEC23A–S1008A axis has not been reported. Our study reveals for the first time an alternative mechanism of autophagy activation by SEC23A in tumor cells, i.e, through the secretory protein pathway. At the mechanistic level of the pro-autophagy activity of S1008A, although we have shown the effect of S100A8 on BECLIN1 expression, the nature of their interaction is not direct. Thus, regulation of BECLIN1 by S100A8 is indirect and merits future investigation.

At last, we verified the clinical relevance of our experimental findings by bioinformatics analysis of the expression of Sec23a and S100A8 and the clinical-pathological associations. The clinical data analysis with TCGA database showed that higher Sec23a and Atg5 expression levels appear to be protective factors and favorable diagnostic (TNM staging) and prognostic (overall survival) markers for SKCM and COAD patients, though they are not independent markers (data not shown). Characterization of the diagnostic and prognostic significance of secreted S100A8 requires collection of blood samples from SKCM and COAD patients that is beyond the scope of the present study.

## Supplementary information

Supplementary figure legends

Figure.S1

Figure.S2

Figure.S3
