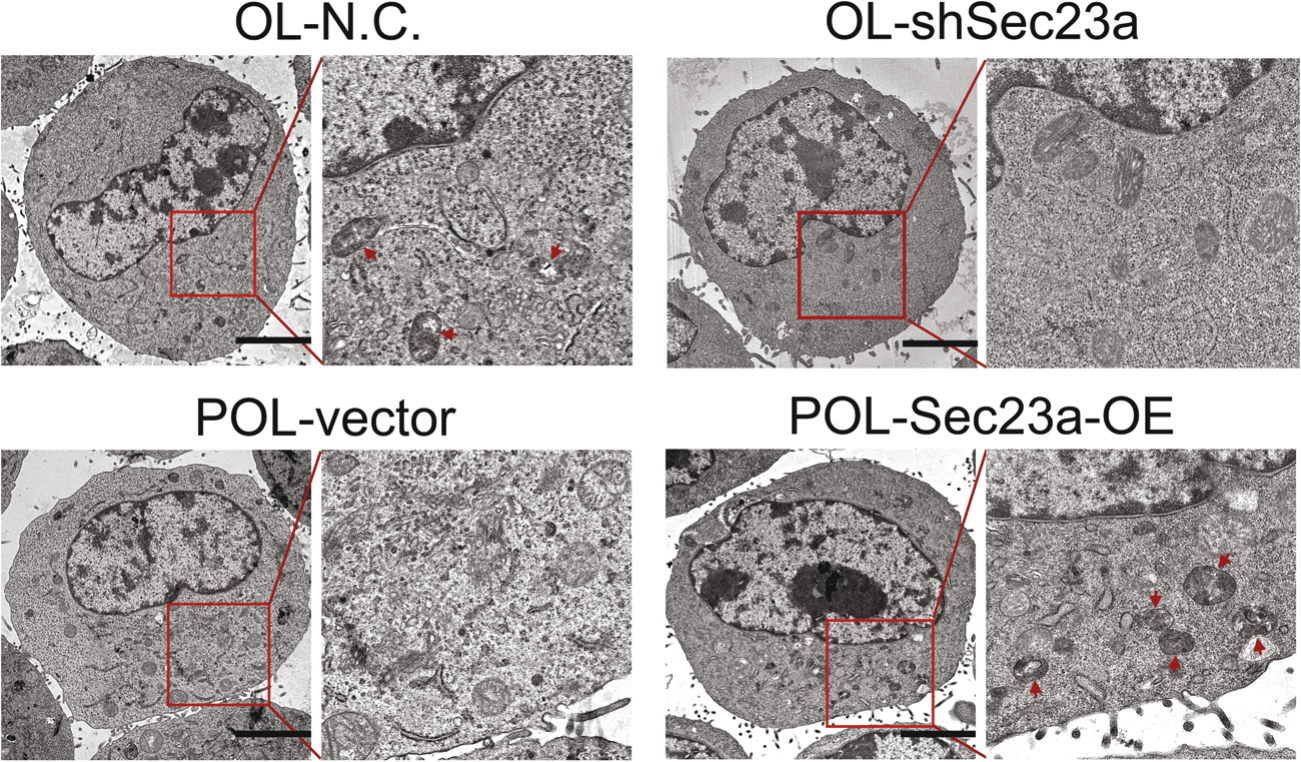# Correction to: S100A8 transported by SEC23A inhibits metastatic colonization via autocrine activation of autophagy

**DOI:** 10.1038/s41419-022-04683-2

**Published:** 2022-03-10

**Authors:** Zhiwei Sun, Bin Zeng, Doudou Liu, Qiting Zhao, Jianyu Wang, H. Rosie Xing

**Affiliations:** 1 Institute of Life Sciences, Chongqing Medical University, Chongqing, China; 2 Laboratory of Translational Cancer Stem Cell Research, Chongqing Medical University, Chongqing, China; 3 State Key Laboratory of Ultrasound Engineering in Medicine Co-Founded by Chongqing and the Ministry of Science and Technology, College of Biomedical Engineering, Chongqing Medical University, Chongqing, China

**Keywords:** Metastasis, Macroautophagy

Correction to: *Cell Death and Disease* 10.1038/s41419-020-02835-w, published online 6 August 2020

The original version of this article unfortunately contained a mistake. After publication of the article, the authors noticed an unintentional error in Fig. 2f. As shown in Fig. 2f, the representative transmission electron microscopy image of “OL-shSec23a” was identical to the image of “POL-vector”. The authors have confirmed that the image of “OL-shSec23a” was mistakenly presented in the original Fig. 2f. This error was caused by unintentionally misplacing the representative image. Nevertheless, this error does not change the conclusion or the discussion of the article. The authors apologize for any inconvenience this error may cause. The corrected Fig. 2f is provided below. The original article has been corrected.