# Supplementary material for: S100A8 transported by SEC23A inhibits metastatic colonization via autocrine activation of autophagy
Source: Cell Death Dis. 2020 Aug 6;11(8):650. doi: 10.1038/s41419-020-02835-w (PMC7435177; doi:10.1038/s41419-020-02835-w)
Supplement: Supplementary file 1 — Supplementary figure legends [file 41419_2020_2835_MOESM1_ESM.docx]

**Figure S1. 3-MA, Baf-A1 and Rapa changed autophagy activity of melanoma cells.** (a) Representative images of LC3B-positive puncta in tumor cells treated with 3-MA, Baf-A1 and Rapa. Scale bars, 20 μm. (b) Transmission electron microscopy of tumor cells treated with 3-MA, Baf-A1 and Rapa. Autolysosomes indicated by arrowheads. Scale bars, 5 μm. (c) Quantification of detectable autolysosomes after 3-MA, Baf-A1 and Rapa treatments, counted on transmission electron microscopy images. ***P*<0.01, ****P*<0.001.

**Figure S2. 3-MA, Baf-A1 and Rapa altered migration and invasion abilities of melanoma cells.** (a and b) Representative images and quantification of transwell migration assay. Scale bars, 60 μm. ***P*<0.01, ****P*<0.001. (c and d) Representative images and quantification of transwell invasion assay. Scale bars, 60 μm. ***P*<0.01, ****P*<0.001.

**Figure S3. Low expression of Sec23a and Atg5 is associated with TNM stages and poor prognosis in human colon adenocarcinoma.** (a-c) Correlation between Sec23a expression and TNM stages in COAD patients using the TCGA database. **P*<0.05. (d-f) Correlation between Atg5 expression and TNM stages in COAD patients using the TCGA database. **P*<0.05. (g and h) Low Sec23a and Atg5 expression levels indicated poor overall survivals for COAD patients using Kaplan-Meier Plots analysis. (g) Correlation between Sec23a and Atg5 expression levels in COAD patients using Spearman test.
